# Improvement of Image Quality Using Hybrid Iterative Reconstruction with Noise Power Spectrum Model in Computed Tomography During Hepatic Arteriography

**DOI:** 10.5334/jbsr.2444

**Published:** 2021-09-14

**Authors:** Hiroshi Hamasaki, Takashi Shirasaka, Yasuhiro Ushijima, Hiroshi Akamine, Yukihisa Takayama, Yuichiro Kubo, Keisuke Ishimatsu, Akihiro Nishie, Toyoyuki Kato

**Affiliations:** 1 Division of Radiology, Department of Medical Technology, Kyushu University Hospital, 3-1-1 Maidashi, Higashi-ku, Fukuoka 812-8582, Japan; 2 Department of Clinical Radiology, Graduate School of Medical Sciences, Kyushu University, 3-1-1 Maidashi, Higashi-ku, Fukuoka 812-8582, Japan; 3 Department of Advanced Imaging and Interventional Radiology, Graduate School of Medical Sciences, Kyushu University, 3-1-1 Maidashi, Higashi-ku, Fukuoka 812-8582, Japan

**Keywords:** CT arteriography, hepatocellular carcinoma, hybrid iterative reconstruction, image quality, noise power spectrum model

## Abstract

**Objectives:**

In CT during hepatic arteriography (CTHA), the addition of a noise power spectrum (NPS) model to conventional hybrid iterative reconstruction (HIR) may improve spatial resolution and reduce image noise. This study aims at assessing the image quality provided by HIR with a NPS model at CTHA.

**Methods:**

This institutional review board-approved retrospective analysis included 26 patients with hepatocellular carcinomas (HCCs) who underwent CTHA. In all acquisitions, images were reconstructed with filtered back projection (FBP), adaptive iterative dose reduction 3D (AIDR), and AIDR enhanced (eAIDR) with the NPS model. Four radiologists analyzed the signal-to-noise ratio (SNR) of HCC nodules and its associated feeding arteries. The radiologists used a semiquantitative scale (–3 to +3) to rate the subjective image quality comparing both the FBP and eAIDR images with the AIDR images.

**Results:**

The feeding arteries’ attenuation was significantly higher in eAIDR compared to AIDR [514.3 ± 121.4 and 448.3 ± 107.3 Hounsﬁeld units (HU), p < 0.05]. The image noise of eAIDR was significantly lower than that of FBP (15.2 ± 2.2 and 28.5 ± 4.8 HU, p < 0.05) and comparable to that of AIDR. The SNR of feeding arteries on eAIDR was significantly higher than on AIDR (34.1 ± 7.9 and 27.4 ± 6.3, p < 0.05). Subjective assessment scores showed that eAIDR provided better visibility of feeding arteries and overall image quality compared to AIDR (p < 0.05). The HCC nodule visibility was not significantly different among the three reconstructions.

**Conclusion:**

In CTHA, eAIDR improved the visibility of feeding arteries associated with HCC nodules without compromising nodule detection.

## Introduction

Transcatheter arterial chemoembolization (TACE) is often the treatment of choice for patients with hepatocellular carcinoma (HCC) [1,2]. In a TACE procedure, computed tomography (CT) hepatic arteriography (CTHA) has been reported to provide the highest detection for HCC [3,4]. However, as super-selective TACE needs to identify and select tumor-feeding arteries that are smaller than the segmental branch, higher-resolution images are required compared to whole-liver TACE [5,6]. In addition, thin-slice images are indispensable for identifying tiny vessels such as tumor-feeding arteries, but their use involves a greater amount of image noise than thick-slice images, and the increased image noise could prevent the visualization of tiny vessels.

The hybrid iterative reconstruction (HIR) with a noise power spectrum (NPS) model is expected to reduce image noise without degrading the spatial resolution [7,8]. We thus speculated that HIR with the NPS model would be useful for CTHA, and we conducted the present study to assess the objective and subjective image quality of HIR with NPS model for CTHA as compared to both filtered back projection (FBP) and HIR without NPS model.

## Materials and Methods

The ethics committee of our institution approved this retrospective study with a waiver for the need for informed consent (No, 2020-29).

### Patients

The subjects were 26 patients (12 males and 14 females) with 66 HCC nodules from our facility with clinically suspected HCC who underwent CTHA. The trial period was from November 2017 to November 2018. The patients’ average age was 72.6 years (range 50–85 years) and their average body weight was 57.5 kg (range 41.2–91.8 kg). All patients underwent pretreatment physical and laboratory examinations, ultrasound, and three-phase dynamic CT or gadoxetic-enhanced magnetic resonance imaging (MRI). Hypervascular HCC was diagnosed based on the patients’ imaging findings in addition to high serum levels of tumor markers. The mean tumor diameter was 9.4 mm (range 4–26 mm).

### CTHA

Each patient’s CTHA was performed with an interventional radiology CT (IVR-CT) system equipped with 320 detector-row CT (Aquilion ONE ViSION edition; Canon Medical Systems, Otawara, Japan). The catheter tip was advanced as close to the tumor as possible. CTHA was performed for the area(s) containing at least one HCC nodule. The CTHA data acquisition began 7–10 s (first phase) and 30 s (second phase) after the initiation of a transcatheter hepatic arterial injection of 10–40 mL of nonionic contrast material (iopamidol, Iopamiron® 150 iodine, 150 mg I/mL; Bayer HealthCare, Osaka, Japan) at a speed of 0.5–2.5 mL/s by an automated power injector. The contrast material was injected until the completion of the scanning of the first phase. The parameters for the scanning were as follows: tube voltage, 120 kV; rotation time, 0.5 s; beam collimation, 80 × 0.5 mm; pitch, 0.81; xyz-axis modulated tube current, 160–700 mA (100–430 mAs tube current-time product) [9].

### Image Reconstruction

Images were reconstructed at 0.5-mm thickness with a 320-mm of field of view. In all acquisitions, FBP and two types of HIR [AIDR 3D mild (AIDR) and AIDR 3D enhanced mild (eAIDR); Canon Medical Systems] with the FC13 kernel were performed. eAIDR is an iterative reconstruction with NPS model that preserves high-frequency noise.

### Clinical Study

Four experienced interventional radiologists (YU, YT, YK, and KI) blinded to the patients’ information retrospectively reviewed the findings obtained by CTHA. HCC was defined as nodules that showed high enhancement accompanied by corona enhancement on CTHA. Corona enhancement was defined as peritumoral contrast enhancement on the second phase of CTHA. The noise index of the scan protocols was optimized for AIDR.

### Quantitative Evaluation

The following parameters were measured on axial images at first phase of CTHA by four readers. 1) The mean CT attenuation of the abdominal aorta and HCC nodules was determined by placing a circular region of interest (ROI). The four readers placed the ROI of the abdominal aorta (ROI_Ao_: mean area 200 mm^2^) in a cross-section that did not include the contrast material and catheter. The readers also identified the feeding arteries and measured their CT attenuation by a single pixel unit measurement. The CT attenuation of feeding arteries was measured five times, and the mean was obtained by averaging these values. 2) Image noise was determined as the standard deviation (SD) of the CT attenuation in ROI_Ao_. We measured three ROIs in three different sequential slices and calculated the mean of the measurements from all ROIs. 3) The signal-to-noise ratio (SNR) of the HCC nodules and feeding arteries was calculated as SNR = the mean CT attenuation/image noise [10].

### Qualitative Evaluation

The FBP and eAIDR were compared with the corresponding AIDR. The four interventional radiologists independently assessed all three series one by one, taking into consideration three qualities: 1) the visibility of the tumor, 2) the visibility of feeding arteries distal to the subsegmental branch, and 3) the overall image quality. A semiquantitative seven-point scale was used: from –3 for inferior (impairing the diagnosis) to +3 for superior (easing the diagnosis); –2 for inferior (probably impairing diagnosis) to +2 for superior (probably easing diagnosis); –1 for slightly inferior (no influence on diagnosis) to +1 slightly superior (no influence on diagnosis); and 0, equal. Image quality scores were recoded individually for each of the four readers, and the overall median image quality score was calculated using the scores from all readers.

### Phantom Experiment

**Figure 1:**
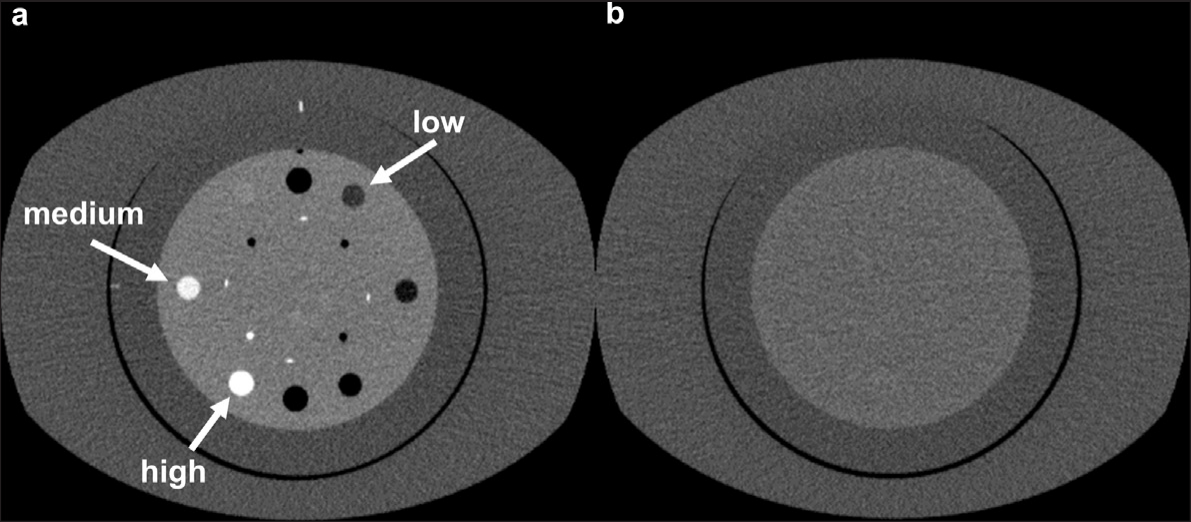
Axial image of the body phantom. A Catphan phantom attached with an oval annulus (25 × 35 cm; 95 cm circumference, The Phantom Laboratory, Salem, NY, USA). a; CTP 404, b; CTP 486.

For normal-sized abdominal models, we used the Catphan 600 phantom (The Phantom Laboratory, Greenwich, NY) with an attached oval annulus (25 × 35 cm; 95 cm circumference). We used two different modules, that of CTP 404 and CTP 486 (***Figure 1***). The scan parameters and reconstructions were processed equivalent to the clinical study described above.

For in-plane spatial resolution, we calculated modulation transfer function (MTF) curves according to the disk methodology [11] from the phantom experiments with three disk-shaped objects with a 12 mm diameter in the CTP 404 module: one object with a high contrast level (Teflon, with 850 HU contrast from the background), one with medium contrast (Delrin, 250 HU contrast), and one with low contrast (polystyrene, 140 HU contrast).

Noise was characterized by calculating the NPS with the CTP 486 module. The NPS was calculated by the radial frequency method with a square ROI of 256 × 256 pixels was placed at the center of each reconstructed image [12]. We also calculated the normalized NPS by using NPS data sets.

These data were analyzed using the software packages CTmeasure 0.97f (Japanese Society of CT Technology, Hiroshima, Japan).

### Statistical Analysis

The mean CT attenuation, image noise, and SNR were tested with Tukey’s honestly significant difference test. Subjective image scores were tested for significance using the Wilcoxon test corrected with the Bonferroni adjustment. Inter-reader reliability was assessed using intraclass correlation coefficient. All statistical analyses were carried out using the statistical software package JMP 15.1.0 (SAS Institute, Cary, NC, USA). A p-value <0.05 was considered signiﬁcant.

## Results

### Quantitative Evaluation

The mean CT attenuation of the feeding arteries was significantly higher in eAIDR compared to those in AIDR (514.3 ± 121.4 and 448.3 ± 107.3 HU, respectively; p < 0.05, ***Table 1***). The mean SNR of feeding arteries in eAIDR was significantly higher than that in AIDR (34.1 ± 7.9 and 27.4 ± 6.3 HU, respectively; p < 0.05). The mean image noise was significantly lower in eAIDR than in FBP (15.5 ± 2.2 and 28.5 ± 4.8 HU, respectively; p < 0.05), which resulted in the SNR on eAIDR being significantly higher than that on FBP (all p < 0.05).

**Table 1:** Quantitative image analysis.


	FBP^†^	AIDR^‡^	EAIDR^§^

CT attenuation (HU)			

Abdominal aorta	69.0 ± 15.7	69.2 ± 15.5	69.5 ± 15.5

Tumor	305.0 ± 109.8	303.1 ± 109.2	305.3 ± 110.3

Feeding artery	493.8 ± 107.3	448.3 ± 104.5	514.3 ± 121.4^**^

Image noise (HU)	28.5 ± 4.8	15.5 ± 1.8^*^	15.5 ± 2.2^*^

SNR^¶^			

Tumor	10.8 ± 4.9	18.2 ± 5.3^*^	18.5 ± 5.5^*^

Feeding artery	16.7 ± 4.2	27.4 ± 6.3^*^	34.1 ± 7.9^*, **^


Data are presented as the mean±standard deviation. ^†^ FBP: ﬁltered back projection, ^‡^ AIDR: adaptive iterative dose reduction 3D, ^§^ eAIDR: AIDR enhanced, ^¶^ SNR: the signal-to-noise ratio. ^*^ indicate statistically significant difference as compared to FBP. ^**^ indicate statistically significant difference as compared to AIDR.

### Qualitative Evaluation

**Figure 2:**
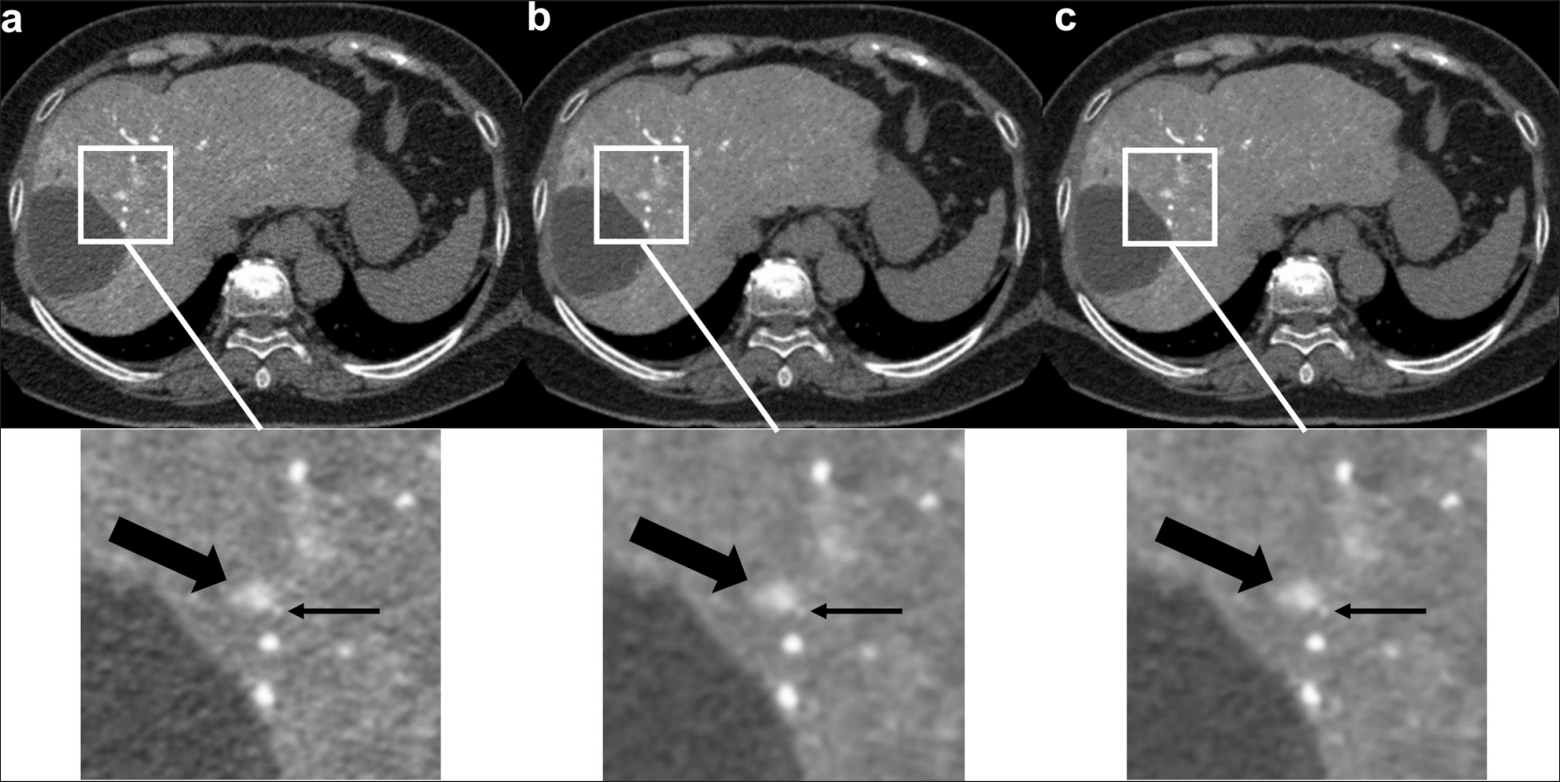
A 73-year-old male with HCC (thick arrow) and its feeding artery (thin arrow). Image quality of three reconstruction methods is shown: a, FBP; b, AIDR, and c, eAIDR. The eAIDR yielded noise reduction and high feeding artery contrast enhancement. FBP: ﬁltered back projection; AIDR: adaptive iterative dose reduction 3D; eAIDR: AIDR enhanced.

eAIDR was superior to AIDR in terms of improved visibility of the feeding arteries (median; +1, p < 0.05) and overall image quality (median; +1, p < 0.05, ***Table 2***). Compared to AIDR, FBP reduced the overall image quality (median; –1, p < 0.05) but did not affect the visibility of the feeding arteries. Tumor visibility was not significantly different among the three reconstructions. The inter-observer agreement ranged from 0.79 to 0.93, indicating high agreement among the four readers. A representative case is shown in ***Figure 2***.

**Table 2:** Subjective image quality scores.


	FBP^†^		EAIDR^‡^	
	
	MEDIAN (MIN, MAX)	VS AIDR^§^ *p* VALUE	MEDIAN (MIN, MAX)	VS AIDR^§^ *p* VALUE

Tumor	0 (–1, 1)	0.45	0 (–1, 1)	0.45

Feeding artery	0 (0, 1)	0.18	1 (0, 2)	0.02^*^

Overall image quality	–1 (–2, 1)	0.01^*^	1 (–1, 1)	0.01^*^


Data are mean subjective image quality ratings regarding the visibility of the tumor, the visibility of its associated feeding arteries, and the overall image quality in comparison with baseline AIDR image rating at 0. ^†^FBP: ﬁltered back projection, ^‡^eAIDR: adaptive iterative dose reduction 3D enhanced, ^§^AIDR: adaptive iterative dose reduction 3D. ^*^ indicate statistically significant difference *(*p < 0.05).

### Phantom Experiment

**Figure 3:**
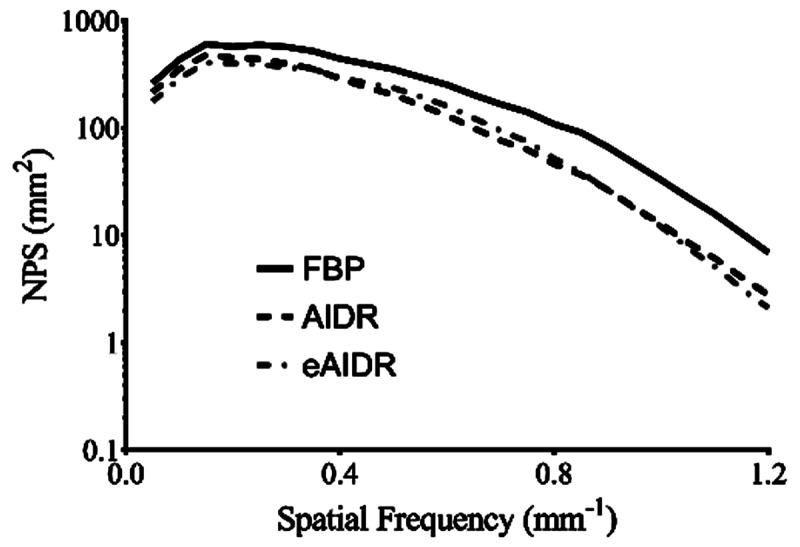
The noise power spectrum curves obtained at different reconstruction. FBP: ﬁltered back projection; AIDR: adaptive iterative dose reduction 3D; eAIDR: AIDR enhanced.

**Figure 4:**
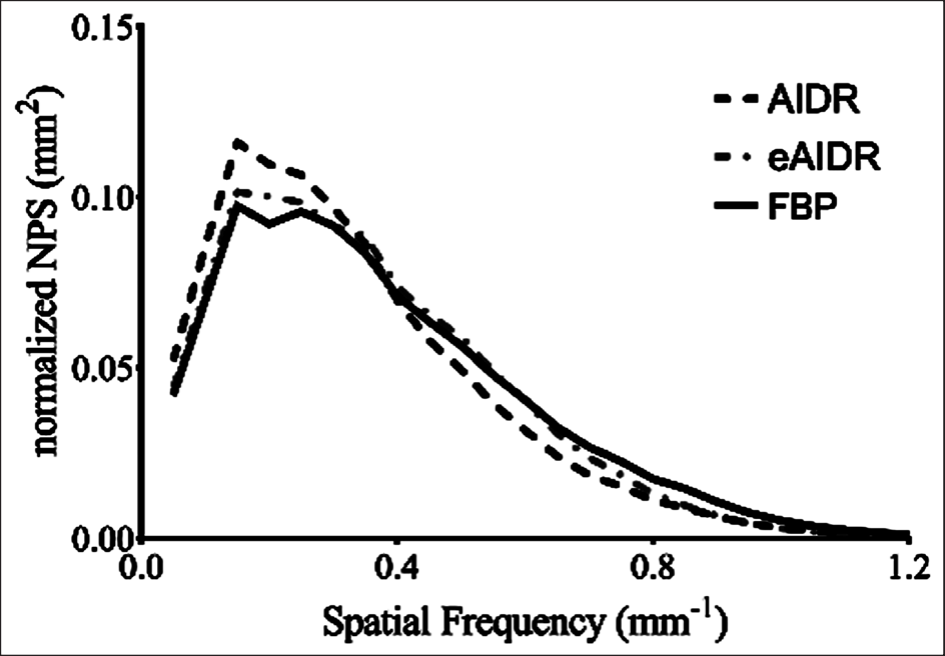
The normalized noise power spectrum curves obtained at different reconstruction. FBP: ﬁltered back projection; AIDR: adaptive iterative dose reduction 3D; eAIDR: AIDR enhanced.

At each contrast level, the spatial frequency at the 10% MTF of the eAIDR was the highest among the three reconstructions, and that of AIDR showed the lowest spatial resolution (***Table 3***). The NPS curves of eAIDR and AIDR were lower than that of FBP over the entire frequency range (***Figure 3***). ***Figure 4*** shows the normalized NPS curves; the normalized NPS curves of eAIDR were similar to those of FBP and both lower than those of AIDR.

**Table 3:** Spatial frequency at 10% MTF^†^ at different contrast levels.


CONTRAST LEVEL	FBP^‡^	AIDR^§^	EAIDR^¶^

High	0.65	0.63	0.80

Medium	0.66	0.62	0.79

Low	0.64	0.63	0.75

cycles/mm.			

Data are means. ^†^MTF: modulation transfer function, ^‡^FBP: ﬁltered back projection, ^§^AIDR: adaptive iterative dose reduction 3D, ^¶^eAIDR: AIDR enhanced.

## Discussion

Our quantitative evaluation of CTHA showed that eAIDR with NPS model reduced the image noise and improved the spatial resolution. The qualitative evaluations demonstrated that eAIDR improved the visibility of the feeding arteries without compromising the detection of HCC nodules. The results of the quantitative evaluation in the phantom experiment were in agreement with the results of the clinical study.

eAIDR was superior to AIDR regarding the visibility of feeding arteries distal to the subsegmental branch. This is because the mean CT attenuation of feeding arteries in eAIDR was significantly higher than that of AIDR, which improved the SNR of the feeding arteries in eAIDR. Therefore, eAIDR is superior to AIDR for visualizing tiny structures on CT arteriography which provides high-contrast images [7].

We also observed that the tumor visibility was not significantly different among the three reconstructions. Since the hypervascular HCC nodules had sufficient contrast on the images, and we speculate that the image noise had a small effect on the tumor visibility. In addition, the minimum size of the HCC nodules was 4 mm, and thus the tumors were large enough to minimize the effect of the differences in spatial resolution. Therefore, the three reconstructions examined herein did not affect the visibility of the tumors.

Compared to the AIDR, the subjective overall image quality was reduced in the FBP and improved in the eAIDR. Since the FBP showed more image noise than the AIDR, we suspect that the overall image quality on FBP was lower than on AIDR. The frequency characteristics of the image noise on eAIDR were similar to those on FBP, and eAIDR showed the same image noise as AIDR; this means that eAIDR can perform denoising without image unnaturalness compared to AIDR. We thus considered that eAIDR images are easier for radiologists to accept than AIDR images.

Two types of iterative reconstruction (IR) methods are used for image reconstruction [i.e., model-based IR (MBIR)] and HIR, and AIDR and eAIDR are classified as HIR. MBIR is superior to HIR in terms of spatial resolution and image noise reduction, but MBIR requires approximately five times more computational time than HIR [13,14]. In addition, CTHA requires thin-slice images for the identification of feeding arteries, and the need for hundreds of images increases the computational time and affects the throughput of the procedure. eAIDR is useful because it can be reconstructed faster than MBIR, allowing images to be displayed faster during angiography procedures.

The present study has a few limitations. First, we did not evaluate the recently released deep learning-based reconstruction (DLR) algorithm. DLR algorithms are expected to help reconstructing images more quickly than MBIR and dramatically reduce image noise without compromising spatial resolution [15,16]. However, the popular IVR-CT does not have the DLR algorithm. Second, the number of patients was relatively small (n = 26), such that our study results require confirmation by future larger studies.

## Conclusion

In conclusion, eAIDR improved the visibility of the feeding arteries associated with HCC nodules on CTHA, without compromising the detection of HCC nodules.
